# Management of children with non-acute abdominal pain and diarrhea in Dutch primary care: a retrospective cohort study based on a routine primary care database (AHON)

**DOI:** 10.1080/02813432.2023.2231054

**Published:** 2023-07-10

**Authors:** Sophie M. Ansems, Marjolein Y. Berger, Elaine Pieterse, Sjaantje Nanne, Gina G. Beugel, Ria P. E. Couwenberg, Gea A. Holtman

**Affiliations:** Department of Primary and Long-term Care, University Medical Center Groningen, Groningen, the Netherlands

**Keywords:** General practice, children, abdominal pain, diarrhea, functional disorders

## Abstract

**Objective:**

To describe the testing, prescription, referral, and follow-up management by general practitioners (GPs) for children presenting with non-acute abdominal pain and/or diarrhea in primary care.

**Design:**

Retrospective cohort study with one-year follow-up.

**Setting:**

Registry data from a Dutch primary care database (AHON) between 2015 and 2019.

**Subjects:**

Children aged 4–18 years old who presented by face-to-face consultation in primary care for non-acute abdominal pain and/or diarrhea (>7 days).

**Main outcome measures:**

We recorded the proportions of children who received (1) diagnostic testing, medicine prescriptions, follow-up consultations, and referrals at their first visit and (2) repeat consultations and referrals by one-year of follow-up.

**Results:**

Among the 2200 children (median age, 10.5 years; interquartile range, 7.0–14.6) presenting to a GP with non-acute abdominal pain and/or diarrhea, most reported abdominal pain (78.7%). At the first visit, GPs performed diagnostic testing for 32.2%, provided a prescription to 34.5%, and referred 2.5% to secondary care. Twenty-five percent of the children had a follow-up consultation within four weeks and 20.8% had a repeat consultation between four weeks and one year. Thirteen percent of the children were referred to secondary care by one year. However, only 1% of all children had documentation of an organic diagnosis needing management in secondary care.

**Conclusion:**

One-third of children received diagnostic testing or a medicine prescription. Few had a follow-up consultation and >10% was referred to pediatric care. Future research should explore the motivations of GPs why and which children receive diagnostic and medical interventions.

## Key points

General practitioners (GPs) often manage children with non-acute abdominal pain and/or diarrhea, which is typically due to a functional gastrointestinal disorder (FGID).

Nearly one-third of all children underwent diagnostic testing at their first visit.

Although recommended by the guideline of the Dutch Society of GPs, we found that only a quarter of children received a follow-up consultation.

Thirteen percent of children were referred to pediatric specialist care by one year.

## Introduction

General practitioners (GPs) face two clinical challenges when presented with children who report non-acute abdominal pain and/or diarrhea. The first is that they might experience diagnostic uncertainty when trying to distinguish organic illnesses (e.g. celiac disease and inflammatory bowel disease) from common functional gastrointestinal disorders (FGIDs) (e.g. functional abdominal pain) that have similar clinical presentations, compounded by access to a limited amount of tests with high diagnostic accuracy [1–3]. Although testing and referring children with FGID may unnecessarily burden the child and result in medicalization [4], underdiagnosing severe organic illnesses might cause delayed treatment and complications [5–9]. The second challenge is that children with FGID often have persistent symptoms, with 50% of those affected still reporting abdominal pain that affects daily activities 12 months after presenting [10]. FGID may also affect the child’s quality of life [11] and school absenteeism [12].

According to the guideline of the Dutch Society of GPs (*Nederlands Huisartsen Genootschap*; NHG), GPs should educate and reassure children with FGID and their parents, and plan a follow-up consultation within two weeks [13]. The follow-up consultation makes the child and parents feel heard and it provides the opportunity to discuss the goal of the treatment again and to answer any questions [13]. If symptoms persist, GPs can refer within primary care systems (e.g. psychotherapy) or to secondary care [13,14]. Although an average GP sees at least 10 children with non-acute abdominal pain and/or diarrhea each year [15,16], we do not know how they handle these challenges. Previous studies have provided some insight into how GPs manage these children [15,17,18], but only in small populations [15,17,18], for acute symptoms [17,18], or focusing on nonspecific abdominal pain [15,16].

To identify how GPs could improve the management of children with non-acute abdominal pain and/or diarrhea, we must understand how they currently manage these children. Therefore, this study aimed to describe the proportion of children with diagnostic testing, prescriptions, follow-up consultations and referrals of all children presenting with non-acute abdominal pain and/or diarrhea in primary care.

## Methods

### Database

We conducted a retrospective cohort study of children presenting with non-acute abdominal pain and/or diarrhea (>7 days), using data from The Academic General Practitioner Development Network Database (AHON; *Academisch Huisarts Ontwikkel Netwerk*) and a one-year follow-up period. Since 2012, this registry database has prospectively collected data from the electronic registration of daily patient care at 57 general practices in the north of the Netherlands. Newly registered patients enter the cohort from their registration date. All Dutch inhabitants are registered with a single general practice at any given time. The AHON database includes the following: International Classification of Primary Care (ICPC) codes [19], diagnostic tests registered according to the NHG guideline [20], drug prescriptions based on Anatomical Therapeutic Chemical Classification (ATC) system codes, contact type (face-to-face consultations, home visits, telephone consultations, e-mail consultations and notes), and pseudonymized contact data documented by the GP as free-text in SOAP (Subjective, Objective, Assessment, Plan) notation [21]. For the selection of our study population and data collection, we employed a combination of automated database queries and manual extraction from free-text documentation. This approach was necessary because certain data were either unavailable or unreliable through the database queries alone. AHON staff were responsible for performing the database queries, while the authors retrieved information from the free-text documentation. Table 1 provides further detail.

**Table 1.** Data collection for patient characteristics and outcomes.

	Moment of assessment		Method of assessment	
	Baseline consultation	One year follow-up	Database query	Hand-searched in free-text documentation^a^
Characteristics				
Gender	X		X	
Age	X		X	
Type of symptoms	X			X
Duration of symptoms	X			X
Final diagnosis	X	X		X
GP management				
Diagnostic tests	X		X	X
Medicine prescriptions	X		X	X
Follow-up consultations	X	X	X	
Referral	X	X		X

^a^Hand-searched in free-text documentation of the baseline consultation for all contacts assigned an inclusion ICPC code during a one-year follow-up period.

The Medical Research Ethics Committee (MREC) of the University Medical Center Groningen (the Netherlands) approved this study (MREC-number: 202100077). All data were pseudonymized in compliance with European Union guidelines on the use of medical data for research. This study is reported according to The Reporting of Studies Conducted using Observational Routinely-collected Health Data (RECORD) statement [22].

### Selection of study population

We included children aged 4–18 years who had a first contact for non-acute abdominal pain (>7 days or unknown duration) and/or non-acute diarrhea (>7 days or unknown duration) between 1 January 2015 and 31 December 2019. Children were excluded if they had a contact assigned D99.06 (celiac disease) or D94 (inflammatory bowel disease) before the start of the study period and if their first contact was not face-to-face (e.g. telephone or e-mail). We have first selected children with a first contact with an inclusion ICPC code (Supplementary Material S1) during the study period. Consequently, we excluded children based on the free-text documentation of this first contact.

### Data collection

The baseline consultation for each child was the earliest face-to-face presentation with non-acute abdominal pain and/or diarrhea. We recorded details of the patient characteristics and GP management outcomes at the baseline consultation and diagnoses, repeat consultations and referrals over a one-year follow-up period.

#### Patient characteristics

Gender and age were retrieved *via* a database query. We manually extracted the type and duration of symptoms, plus the (differential) diagnosis, from free-text-documentation for the baseline consultation, following a standard operating procedure (Supplementary Material S2). Similarly, the final diagnosis was extracted from free-text-documentation based on the most recently documented diagnosis within the one-year follow-up. We categorized the final diagnosis into FGID, organic disease manageable in primary care (e.g. gastroenteritis), organic disease requiring management in secondary care (e.g. celiac disease), and unspecified, as detailed in Supplementary Material S3.

#### GP management

##### Diagnostic testing and prescriptions: baseline consultation

We selected diagnostic tests within two weeks after baseline *via* a query in the diagnostic intervention database. The query included clinically relevant tests for children with abdominal pain and/or diarrhea given the broad differential diagnosis, determined by the first two authors (former pediatric resident and GP) (Supplementary Material S4). Relevant tests included blood tests, urinalysis, urine dip slide, and fecal analysis; however, we did not include imaging or fecal- and urine cultures because they could not be accessed anonymously in the AHON database. Also, because C-reactive protein (CRP) is available as a point-of-care-test and may not be registered in the diagnostic intervention database, we manually searched for evidence of CRP testing in the free-text documentation.

Next, we used ATC codes to identify prescriptions within two weeks after the baseline consultation *via* a database query. Based on the first and second authors’ clinical expertise, we distinguished medicine typically prescribed for non-acute abdominal pain and/or diarrhea (e.g. laxatives, Supplementary Material S4) from medicine that could have been prescribed for other symptoms (e.g. analgesics, Supplementary Material S4). For the latter, we reviewed the free-text documentation to check whether prescriptions were for the non-acute abdominal pain and/or diarrhea or for a different medical problem. We removed prescriptions from the dataset if prescribed for a problem other than abdominal pain and/or diarrhea and when administrative errors resulted in the same drug being prescribed twice on the same day or on two consecutive days.

##### Follow-up and repeat consultations at baseline consultation and one year

By database query, we included all telephone, face-to-face, or e-mail contacts within one year after baseline consultation as follow-up or repeat consultations if assigned an inclusion ICPC codes. Consequently, all unknown contact types were dropped from the dataset. The NHG guideline recommends planned follow-up within two weeks after the first presentation [13]. Allowing for a two week delay due to planning difficulties, we considered follow-up consultations as those within four weeks after the baseline consultation and repeat consultations as those between four weeks and one year.

##### Referrals at baseline consultation and one year

The AHON database does not consistently register referrals, which we defined as those within the primary care system or to secondary care. Research staff manually extracted referral data from the free-text-documentation at the baseline consultation and one year follow-up, according to a standard operating procedure.

### Statistical analysis

To investigate the management of children with non-acute abdominal pain and/or diarrhea by GPs, we describe the proportions of included children with the following outcomes from the baseline consultation (at least one per outcome): diagnostic tests, medicine prescriptions, follow-up consultations, or referral. Similarly, we describe the proportions of children with at least one repeat consultation and at least one referral between four weeks and one year. For referrals, we also describe the proportion of children with at least one referral throughout the whole study period and the time between first referral and the baseline consultation. Normally distributed continuous data are presented as means and standard deviations, while non-normally distributed continuous data are presented as medians and interquartile ranges (IQR). We used IBM SPSS for Windows, Version 25 (IBM Corp., Armonk, NY, USA) to analyze the data, but refrained from analysis beyond providing descriptive statistics.

## Results

### Study population

**Figure 1.**
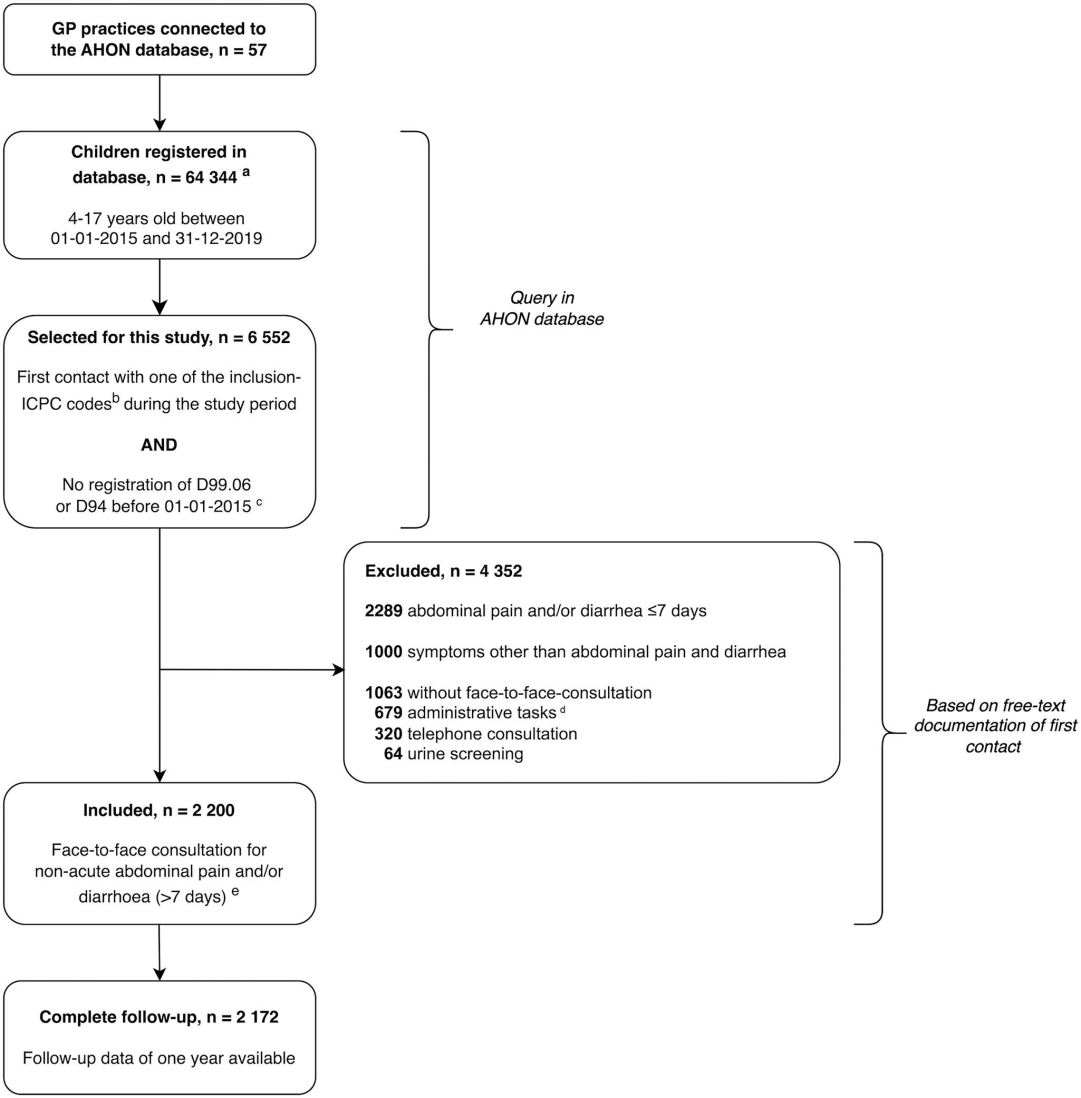
Flow chart of patient inclusion. ICPC: International Classification of Primary Care. ^a^Not reliable for prevalence calculation due to invalid estimation of numerator. ^b^Inclusion ICPC codes are shown in Supplementary Material S1. ^c^ICPC code D99.06: celiac disease; ICPC code D94: inflammatory bowel disease. ^d^These include correspondence with other healthcare professionals, processing test results, and prescribing medication. ^e^Double inclusions due to duplicate pseudonymized identification numbers were removed (n = 11).

Figure 1 shows the selection of our study population. We included 2200 children who presented with a first face-to-face consultation for non-acute abdominal pain and/or diarrhea (Figure 1). These consultations involved 100 different GPs working in 57 general practices (median; 21.0 consultations/GP, IQR; 4.3–33.5).

Most included children were female (58.9%) and presented with abdominal pain (78.7%) (Table 2). Only 28 children (1.3%) had available follow-up times less than one year (median 155 days; IQR 57–194). By one year, GPs diagnosed 1836 children (83.5%) with FGID and 214 (9.7%) with an organic disease manageable in primary care (Table 3). Overall, 23 children (1.0%) received a diagnosis of organic disease requiring management in secondary care (Table 3).

**Table 2.** Characteristics of children presenting with non-acute abdominal pain and/or diarrhea in general practice.

Characteristic	*n* = 2200
Male, n (%)	905 (41.1)
Age, median years (IQR)	10.4 (7.0–14.6)
Symptoms, n (%)	
Abdominal pain	1732 (78.7)
Diarrhea	173 (7.9)
Both	295 (13.4)
Duration of symptoms, n (%)	
>1 week < 2 months	846 (38.5)
≥2 months	721 (32.8)
Unclear	633 (28.8)
Diagnosis according to GP during baseline consultation, n (%)^a^	
Abdominal pain, cause unknown	772 (30.5)
Constipation	545 (21.5)
Irritable bowel syndrome	222 (8.7)
Functional abdominal pain	212 (8.4)
Gastrointestinal infection	204 (8.1)
Diarrhea, cause unknown	201 (7.9)
Gastritis / heartburn	80 (3.2)
Urinary tract infection	72 (2.8)
Suspicion of celiac disease	47 (1.9)
Gynaecological diagnosis	44 (1.7)
Food intolerance / allergy	24 (0.9)
Suspicion of appendicitis	20 (0.8)
Suspicion of inflammatory bowel disease	19 (0.8)
Other	43 (1.7)
No diagnosis	26 (1.0)

^a^Each child can have multiple diagnoses, as documented by the GP. Denominator = 2531.

**Table 3.** Final diagnosis in 2200 children presenting in general practice with non-acute abdominal pain and/or diarrhea.

Diagnosis received by one year of follow-up, *n* = 2200	n (%)
Functional gastrointestinal disorders	
Abdominal pain with unknown cause	702 (31.9)
Constipation	467 (21.2)
Irritable bowel syndrome or functional abdominal pain	386 (17.5)
Diarrhea with unknown cause	153 (7.0)
Multiple FGIDs^a^	77 (3.5)
Functional dyspepsia	51 (2.3)
Organic diseases manageable in primary care	
Gastroenteritis	158 (7.2)
Urinary tract infection	25 (1.1)
Gastritis	11 (0.5)
Lactose intolerance	7 (0.3)
Dysmenorrhea	7 (0.3)
Vaginitis	2 (0.1)
Pregnancy	1 (0.1)
Pelvic inflammatory disease	1 (0.1)
Respiratory infection	1 (0.1)
Mesenteric lymphadenitis	1 (0.1)
Organic diseases requiring management in secondary care	
Inflammatory bowel disease	8 (0.4)
Celiac disease	6 (0.3)
Food allergy	3 (0.1)
Endometriosis	1 (0.1)
Cholelithiasis	1 (0.1)
Anterior cutaneous nerve entrapment syndrome	1 (0.1)
Ovarian torsion	1 (0.1)
Hyperthyroidism	1 (0.1)
Umbilical hernia	1 (0.1)
Unspecified	
Without referral	73 (3.3)
Referred to secondary care	54 (2.5)

^a^Combination of constipation and/or irritable bowel syndrome and/or functional abdominal pain and or functional dyspepsia.

### Diagnostic testing–baseline consultation

GPs performed diagnostic testing in 709 children (32.2%, Table 4). Due to the large heterogeneity in the 41 different tests used (Supplementary Material S5), we present the results for only the 10 most frequently performed tests in Table 4. The most common tests were CRP (*n* = 422, 19.2%), leukocyte count (*n* = 382, 17.4%), and hemoglobin (*n* = 367, 16.7%) (Table 4). Children who underwent diagnostic testing complete a median of 4 tests (IQR, 1–7, not in table).

**Table 4.** GP management at the baseline consultation and during follow-up among 2200 children.

	Baseline consultation	One year follow-up
**Children with ≥ 1 diagnostic test, n (%)**	**709 (32.2)**	**NA**
C-reactive protein	422 (19.2)	
Leukocytes	382 (17.4)	
Hemoglobin	367 (16.7)	
Creatinine	246 (11.2)	
Urinalysis^a^	244 (11.1)	
Glucose	206 (9.4)	
Erythrocyte sedimentation rate	202 (9.2)	
Thyroid stimulating hormone	201 (9.1)	
Thrombocytes	179 (8.1)	
Immunoglobulin A / anti-tissue transglutaminase	140 (6.4)/126 (5.7)	
**Children with ≥ 1 medicine prescription, n (%)**	**760 (34.5)**	**NA**
Laxatives	597 (27.1)	
Drugs for acid related disorders	79 (3.6)	
Anti-infective agents	36 (1.6)	
Analgesics	35 (1.6)	
Diarrhea and vomiting inhibitors	29 (1.3)	
Antispasmodics	22 (1.0)	
Contraception	16 (0.7)	
Other	20 (0.9)	
**Children with ≥ 1 (follow-up) consultation, n (%)**	**552 (25.1)**	**457 (20.8)**
Face-to-face consultation, n (%)	399 (18.1)	386 (17.5)
Only telephone or e-mail consultation, n (%)	153 (6.9)	71 (3.2)
Number of follow-up consultations (median, IQR)	1 (1–2)	2 (1–3)
**Children with ≥ 1 referral, n (%)**	**79 (3.6)**	**278 (12.6)**
To secondary care	55 (2.5)	231 (10.5)^c^
Within primary care systems^b^	23 (1.0)	54 (2.5)^d^
Unknown	1 (0.05)	4 (0.2)
Time between baseline consultation and first referral, weeks (median, IQR)	NA	6.9 (2.0–22.6)

^a^Urinalysis: chemical urine tests in the general practice.

^b^Referrals within primary care systems (e.g. dietician, physical therapist, or psychotherapist).

^c^Four children were referred to secondary care during the baseline consultation.

^d^One child was referred within primary care during the baseline consultation.

### Drug prescriptions–baseline consultation

GPs prescribed medication to 760 children (34.5%), with the majority receiving laxatives (78.6%, Table 4). However, they only prescribed two or more drugs to 94 children (4.3%, not in table).

### Follow-up and repeat consultations–baseline consultation and one-year

A quarter of the children (*n* = 552) had a follow-up consultation within four weeks of the baseline consultation, and 399 children (72.3%) of these had at least one face-to-face consultation (Table 4). In the period between four weeks and one year after the baseline consultation, 457 children (20.8%) had a repeat consultation for gastrointestinal symptoms (Table 4).

### Referrals–baseline consultation and one-year

GPs referred 79 children (3.6%) at the baseline consultation, of whom 55 (69.6%) went to secondary care and 23 (29.1%) to primary care systems (Table 4). From baseline consultation to one year, they subsequently referred a further 278 children (12.6%) (Table 4), of whom 231 (83.1%) went to secondary care.

Throughout the study period (including baseline consultation and over the course of one-year follow-up), GPs referred 352 children (16.0%) at least once. Most children were referred within 16 weeks of the baseline consultation (median 3.2 weeks, IQR 0.2–15.8, not in table). Throughout the study period, seventy-six children were referred at least once within primary care systems (3.5%) and 282 (12.8%) were referred at least once to secondary care. Among the 721 children with symptoms ≥2 months, 133 children (18.4%) were referred at least once to secondary care throughout the study period (not in table).

## Discussion

### Statement of principal findings

In this cohort of children aged 4–18 years presenting with non-acute abdominal pain and/or diarrhea in primary care, 32% of children received diagnostic testing and 34% of children received a medication prescription during the initial presentation. GPs only followed up a quarter of all children within four weeks. Over the one-year follow-up period, 13% of children obtained referrals to secondary care and 4% obtained referrals within the primary care system.

### Strengths and weaknesses of the study

The AHON database benefits from containing data for many patients, allowing the identification of sufficient case numbers in a low incidence setting. We believe this is the largest study sample used to describe the management of children with non-acute gastrointestinal symptoms by GPs in primary care [15,17,18]. However, the use of registration data imposes important limitations.

First, challenges exist when selecting the proper patient population retrospectively. We addressed this by reviewing the GP’s documentation about the type and duration of symptoms after selecting children based on ICPC codes, thereby ensuring that our study population comprised only children with non-acute abdominal pain and/or diarrhea. Due the large group of children with non-acute abdominal pain and/or diarrhea presenting at 100 different GPs, we expect our findings to be generalizable to the GP’s management of children with non-acute abdominal pain and/or diarrhea in The Netherlands. GPs in other countries with gatekeeping systems, such as the United Kingdom and Norway [23], are likely to treat similar patient populations to those in our study. Although their management approaches may vary due to different guidelines, they might still find value in comparing their own practices to our findings. Second, the validity of our findings depends on the quality of registration for the ICPC codes, tests, prescriptions, and referrals. Because registration is intended to support daily practice rather than research, the AHON database probably contains missing values, inconsistent coding, and under-reporting. This may have resulted in an underestimation of the proportions of diagnostic tests, prescriptions, follow-up consultations and referrals. We partially accounted for this by supplementing the data from standard database queries with text from free-text documentation. Additionally, we may have overlooked some severe final organic diagnoses due to lack in reporting in the GP records, leading to misclassification bias [24].

Lastly, we only described the GP’s management in the complete group of children, without identifying the subgroups that received specific diagnostic or medical interventions (e.g. what children received laxative treatment). Further research should address this.

### Findings in relation to other studies and their implications

Although similar studies on the primary care management of children with chronic gastrointestinal symptoms have been performed [15,17,18], it is difficult to compare our results because these studies used different follow-up periods and definitions of chronic gastrointestinal symptoms.

We found a diagnostic testing rate of 32% during baseline consultation, which contrasts with a rate of 23% in a Dutch cohort from 2004 to 2006 [17]. This implies that diagnostic testing by Dutch GPs has not decreased since implementing the NHG guideline in 2012 [13]. The guideline recommends testing tissue transglutaminase IgA (tTGA) and total serum IgA for suspected celiac disease and testing hemoglobin, leukocytes and ESR for suspected inflammatory bowel disease (IBD). We found large heterogeneity in the diagnostic tests used. The five most frequently used tests were CRP, leukocyte count, hemoglobin, creatinine and urinalysis. Although recommended by the NHG guideline, leukocyte count and hemoglobin have low or unknown diagnostic value for excluding inflammatory bowel disease (low sensitivity) in children in primary care [1]. Fecal calprotectin may serve as a non-invasive alternative test with high sensitivity and specificity for inflammatory bowel disease in children in primary care [25,26]. However, the NHG guideline does not recommend this test due to insufficient evidence, which may explain its infrequent use (0.32% of children) by GPs in our study. We are currently investigating whether fecal calprotectin testing in primary care changes the GP’s management and improves patient outcomes in a randomized controlled trial [27]. tTGA is the most appropriate test for excluding celiac disease [13,28], but was only used in 6% of children. The frequent use of CRP, although not recommended by the NHG guideline [13], likely reflects a combination of its availability as a point-of-care test in most GP practices and its use to assess children with chronic abdominal pain in secondary care [29]. It’s worth noting that we cannot determine whether diagnostic testing was overused or underused in our study because we lack information on the characteristics (e.g. severity of symptoms or alarm symptoms) of the children who underwent testing.

We found a prescription rate of 34% during baseline consultation, which is lower than the 48% in all pediatric consultations in Dutch primary care [30]. In our study, nearly 80% of all prescriptions were for laxatives. Laxatives indeed are among the most common prescribed medications in children in Dutch primary care [30]. The NHG guideline recommends prescribing laxatives when there are clear signs and signals of constipation, but discourages this practice in cases of functional abdominal pain or irritable bowel syndrome because no research supports their use in these patient groups [13,14]. However, our research did not cover how many children without signs and signals of constipation received laxative treatment, necessitating further studies to address this topic. The actual laxative use might even be higher, because laxatives are also available as over-the-counter medication.

We expected higher follow-up consultation rates because this is a non-invasive way to provide reassurance, answer questions, and monitor symptoms, as recommended by the NHG guideline [13]. In the current study, however, only 18% of children had a face-to-face follow-up consultation within four weeks of their first presentation. As our study used registration data, we cannot ascertain whether the absence of follow-up consultations was due to symptom resolution or a lack of active follow-up from the GP. Based on the responses of questionnaires, a prospective cohort study revealed that 50% of children with abdominal pain continued to have symptoms that negatively impacted their daily function after one year of initial presentation in primary care [10]. We expect this study’s population to be comparable to ours, as they enrolled children with abdominal pain who sought care from Dutch GPs [10]. Other studies showed that children with chronic abdominal pain continue to have symptoms later in life [31,32], suffer from school absenteeism [12], anxiety [12,33] and depression [33]. Follow-up could help the GP to develop a good doctor-patient relationship [34] and offer effective continuity of care, two important principles required for the often long-term care of these children [35]. A recent Norwegian interview study confirmed that GPs find it important to build a good relationship with (parents of) children with FGID [36]. However, time constraints could play a role in the GP’s decision to plan a follow-up consultation, or the four-week window might be too short for them to consider a follow-up of medical benefit

The percentage of referrals to secondary care during the first face-to-face contact in this study (2.5%) was similar to that reported by Gieteling et al. (3%) [15], but lower than the finding of Spee et al. (10%) [17]. The inclusion of children with acute abdominal pain by Spee et al. a population in which the GP wants to exclude severe causes rapidly, could explain the latter. Furthermore, children participating in that study had to provide informed consent, contrasting with the requirements in our study and that conducted by Gieteling et al. [15]. Given the low a priori probability of organic disease and the possible negative effects of referrals in children with non-acute abdominal pain and/or diarrhea [4], the referral rate of 13% to secondary care seems high. A recent Dutch report about low-value care listed pediatrician referral for non-somatic abdominal pain as a high priority for de-implementation [37]. Although GPs might have referred children for other reasons, such as the multidisciplinary treatment of FGID in secondary care, GPs and other primary health care providers could also deliver this care.

## Conclusion and implications

In conclusion, this study highlights that in Dutch primary care, approximately one-third of children presenting with non-acute abdominal pain and/or diarrhea undergo diagnostic testing or are prescribed medication during their initial visit, and few receive active follow-up. More than ten percent of children receives a referral to pediatric specialist care. To better comprehend the factors that underlie these management decisions, future research should explore the motivations of GPs why and which children receive diagnostic and medical interventions.

## Supplementary Material

Supplemental Material
