# Supplementary material for: Management of children with non-acute abdominal pain and diarrhea in Dutch primary care: a retrospective cohort study based on a routine primary care database (AHON)
Source: Scand J Prim Health Care. 2023 Jul 10;41(3):267–75. doi: 10.1080/02813432.2023.2231054 (PMC10478593; doi:10.1080/02813432.2023.2231054)
Supplement: Supplemental Material [file IPRI_A_2231054_SM9668.docx]

Supplementary Material S1. List of Inclusion *International Classification of Primary Care (ICPC)* codes

| **ICPC code** | **Label** |
| --- | --- |
| D01 | Abdominal pain/cramps general |
| D02 | Abdominal pain epigastric |
| D06 | Abdominal pain localized other |
| D11 | Diarrhea |
| D12 | Constipation |
| D16 | Rectal bleeding |
| D18 | Change feces/bowel movements |
| D25 | Abdominal distension |
| D27 | Fear of digestive disease other |
| D29 | Digestive symptom/complaint other |
| D73 | Gastroenteritis presumed infection |
| D93 | Irritable bowel syndrome |
| D94 | Chronic enteritis/ulcerative colitis |
| D99.06 | Celiac disease |
| D99.07 | Lactose intolerance |

Supplementary Material S2. Standard Operating Procedure extracting type and duration of symptoms, differential diagnoses and referral

1. **Type of symptoms during baseline consultation**

| **Type of symptoms** |  |
| --- | --- |
| Abdominal pain | If the presenting symptom is abdominal pain |
| Diarrhoea | If the presenting symptom is diarrhoea |
| Abdominal pain and diarrhoea | If the child has both abdominal pain and diarrhoea |

1. **Duration of symptoms during baseline consultation**

| **Duration of symptoms** |  |
| --- | --- |
| ≥ 2 months | **If symptoms have existed for 2 months or longer**  For example:  *‘Since half a year’ / ‘Lately’ / ‘Regularly’ / ‘Chronic’ / ‘Had to be picked up from school all the time’ / ‘Complains of abdominal pain often’ / ‘Often abdominal pain, this time for 3 days’* |
| >1 week <2 months | **If symptoms have existed for less than 2 months but longer than 1 week**  In case of a specific time indication  ‘*Since 2 weeks*’ / ‘*Since september*’ / *‘Since the start of the new school year’* |
| Unclear | **Symptoms without time indication** |

1. **Referral for the gastrointestinal symptoms**

**2.1 Was the child referred for chronic abdominal pain and/or diarrhoea within 1 year after the baseline consultation?**

***This only concerns referrals for chronic abdominal pain and/or diarrhoea!***

| Yes | The child was referred for chronic abdominal pain and/or diarrhoea within 1 year after the baseline consultation. Go to 2.2 |
| --- | --- |
| No | There was no referral. Go to 3.1 |

**2.2 How many times was the child referred within 1 year after baseline consultation?**

| **1** |
| --- |
| **2** |
| **3** |
| **4** |
| **5** |

**Fill in for each referral:**

**2.3 The date of referral**

| Date of referral | dd/mm/yyyy |
| --- | --- |

**2.4 Where is the child referred to?**

| Pediatrician |
| --- |
| Pediatric gastroenterologist |
| Gastroeneterologist |
| Gynaecologist |
| Psychiatrist |
| Emergency Department |
| Psychotherapy |
| Physiotherapy |
| Dietician |
| Other: ________________ |

**3. Diagnoses**

**3.1 How often was a (differential) diagnosis documented within 1 year after baseline consultation?**

This only concerns (differential) diagnoses for the chronic abdominal pain and/or diarrhoea.

| **1** |
| --- |
| **2** |
| **3** |
| **4** |
| **5** |

**Fill in for each documented diagnosis:**

**3.2 What is the date of documentation?**

|  | dd/mm/yyyy |
| --- | --- |

**3.3 What is the (differential) diagnosis (multiple responses possible)?**

| **Functional Gastrointestinal Disorder (FGID)** | **Examples of GP’s documentation** |
| --- | --- |
| Functional constipation | *‘Constipation’ / ‘constipated’* |
| Irritable bowel syndrome | *‘Irritable bowel syndrom’ / ‘IBS’ / ‘spastic colon’* |
| Functional abdominal pain | *‘Stress’ / ‘functional’ / ‘functional abdominal pain’* |
| Abdominal pain with unknown cause | *‘Recurrent abdominal pain’ / ‘abdominal pain with unknown cause’ / ‘chronic abdominal pain’ / ‘abdominal pain’* |
| Diarrhoea with unknown cause | *‘Chronic diarrhoea’ / ‘diarrhoea with unknown cause’ / ‘Diarrhoea’* |
| **Organic** |  |
| Gastroenteritis | *‘Gastroenteritis’/ ‘GEit is’/ Gastrointestinal infection’/ ‘stomach bug’* |
| Urinary tract infection | *‘Urinary tract infection’ / ‘UTI’* |
| Inflammatory Bowel Disease (IBD), go to 4.2 | *‘Crohn’s disease’ / ‘Ulcerative colitis’ / ‘IBD’* |
| Celiac disease, go to 4.3 | *‘Gluten intolerance’ / ‘gluten allergy’ / ‘celiac disease’* |
| Gastritis | *‘Gastritis’ / ‘heart burn’ / ‘acid reflux’/ ‘Helicobacter pylori’* |
| Lactose intolerance | *‘Lactose intolerance’* |
| Food allergy | *‘Food allergy’* |
| Dysmennorhea | *‘Dysmennorhea’* |
| Appendicitis | *‘Appendicitis’* |
| **Other: _____________________** | All diagnoses that do not belong to one of the categories above |

**4.2 Suspected or confirmed IBD?**

| **Suspicion** | If the diagnosis is not confirmed by a medical specialist(yet) |
| --- | --- |
| **Confirmed by medical specialist** | The medical specialist diagnosed the child with inflammatory bowel disease (IBD) (as documented in correspondence from the medical specialist to the GP) |
| **Unclear** |  |

**4.2 Suspected or confirmed celiac disease?**

| **Suspicion** | If the diagnosis is not confirmed by a medical specialist(yet) |
| --- | --- |
| **Confirmed by medical specialist** | The medical specialist diagnosed the child with celiac disease (as documented in correspondence from the medical specialist to the GP) |
| **Unclear** |  |

Supplementary Material S3. Final diagnosis categories and definitions

| **Final diagnosis** | **Definitions** |
| --- | --- |
| **Functional gastrointestinal disorders** | |
| Abdominal pain with unknown cause | 1. The last documented diagnosis of the GP is “abdominal pain, cause unknown” / “abdominal pain.”  OR  2. The child’s presenting symptom is abdominal pain, and the GP did not document any (differential) diagnosis during 1-year follow-up. |
| Constipation | 1. The last documented diagnosis of the GP is “constipation.”  OR  2. The GP did not document any (differential) diagnosis during 1-year follow-up but during the last consultation the GP prescribed laxatives. |
| Irritable Bowel Syndrome/ Functional Abdominal Pain | 1. The last documented diagnosis of the GP is “functional abdominal pain” / “stress related” / “psychosomatic.”  OR  2. The last documented diagnosis of the GP is “irritable bowel syndrome” / “spastic bowel.”  OR  3. A combination of 1 and 2. |
| Diarrhea with unknown cause | 1. The last documented diagnosis of the GP is “diarrhea, cause unknown” / “diarrhea.”  OR  2. The child’s presenting symptom is diarrhea, and the GP did not document any (differential) diagnosis during 1-year follow-up. |
| Multiple FGIDs | The last documented diagnosis of the GP is a combination of the definitions for “constipation” and “irritable bowel syndrome/ functional abdominal pain.” |
| Functional dyspepsia | The last documented diagnosis of the GP is “functional dyspepsia” / “heartburn” / “reflux” / “stomach” / “pyrosis.” |
| **Organic disorders manageable in primary care** | |
| Gastroenteritis | 1. The last documented diagnosis of the GP is “gastroenteritis” / “stomach flu.”  OR  2. The GP did not document any (differential) diagnosis during 1-year follow-up but during the last consultation, a fecal culture showed a positive finding.  OR  3. The GP did not document any (differential) diagnosis during 1-year follow-up but during the last consultation, the GP prescribed anti-infective agents (such as metronidazole) indicating gastroenteritis. |
| Gastritis | 1. The last documented diagnosis of the GP is “gastritis” or “*Helicobacter pylori*”  OR  2. The GP did not document any (differential) diagnosis during 1-year follow-up but during the last consultation, the GP prescribed a proton pump inhibitor |
| Urinary tract infection | 1. The last documented diagnosis of the GP is “urinary tract infection.”  OR  2. The GP did not document any (differential) diagnosis during 1-year follow-up but during the last consultation, a urine culture showed a positive finding  OR  3. The GP did not document any (differential) diagnosis but during the last consultation, the GP prescribed antibiotics indicating a urinary tract infection. |
| Lactose intolerance | The last documented diagnosis of the GP is “lactose intolerance.” |
| Dysmenorrhea | The last documented diagnosis of the GP is “dysmenorrhea” / “pain during periods” / “related to cycle.” |
| Vaginitis | The last documented diagnosis of the GP is “vaginitis” |
| Pregnancy | The last documented diagnosis of the GP is “pregnancy” and this is confirmed by a positive pregnancy test. |
| Pelvic Inflammatory Disease | The last documented diagnosis of the GP is “pelvic inflammatory disease” and this is confirmed by a positive test for sexually transmitted diseases. |
| Respiratory infection | The last documented diagnosis of the GP is “respiratory infection.” |
| Mesenteric lymphadenitis | The last documented diagnosis of the GP is “mesenteric lymphadenitis” and this is confirmed by a positive finding on an abdominal ultrasound. |
| **Organic disorders requiring management in secondary care** | |
| Inflammatory Bowel Disease | The last documented diagnosis of the GP is “inflammatory bowel disease” and this is confirmed by a medical specialist. |
| Celiac disease | The last documented diagnosis of the GP is “celiac disease” after this is confirmed by a medical specialist. |
| Food allergy | 1. The last documented diagnosis of the GP is “food allergy” and this is confirmed by a positive result on a food allergy test.  OR  2. The last documented diagnosis of the GP is “food allergy” and this is confirmed by a medical specialist. |
| Endometriosis | The last documented diagnosis of the GP is “endometriosis” and this is confirmed by a medical specialist. |
| Cholelithiasis | The last documented diagnosis of the GP is “cholelithiasis” and this is confirmed by a medical specialist. |
| Anterior cutaneous nerve entrapment syndrome (ACNES) | The last documented diagnosis of the GP is “ACNES” and this is confirmed by a medical specialist. |
| Ovarian torsion | The last documented diagnosis of the GP is “ovarian torsion” and this is confirmed by a medical specialist. |
| Hyperthyroidism | The last documented diagnosis of the GP is “hyperthyroidism” and this is confirmed by a medical specialist. |
| Umbilical hernia | The last documented diagnosis of the GP is “umbilical hernia” and this is confirmed by a medical specialist. |
| **Unspecified** | |
| Without referral | The last documented differential diagnosis of the GP entails multiple diagnoses belonging to different diagnosis categories.  AND  The child is not referred to secondary care.  *Example: Differential diagnosis of GP is combination of urinary tract infection and constipation.* |
| Referred to secondary care | The last documented differential diagnosis of the GP entails multiple diagnoses belonging to different diagnosis categories.  OR  The last documented differential diagnosis of the GP entails (a) suspicion(s) of organic disorder(s) requiring management in secondary care.  AND  The child is referred.  AND  There is no information available about the medical specialist’s diagnosis after referral.  *Example 1: The GP suspects celiac disease and refers the child to the pediatrician. However, the GP did not document the final diagnosis of the pediatrician.* |

Supplementary Material S4. List of included diagnostic tests and prescriptions

| **Diagnostic tests** |
| --- |
| **Blood tests** |
| **Leukocytes**  Eosinophils  Monocytes  Basophils  Leukocytes differentiation |
| **Hemoglobin**  Mean corpuscular volume  Erythrocytes  Hematocrit  Mean corpuscular hemoglobin  Mean corpuscular hemoglobin concentration  Red blood cell distribution width  Anemia diagnostics |
| **Thrombocytes**  Shape abnormalities thrombocytes |
| **C-reactive protein**  C-reactive protein point-of-care-test |
| **Creatinine** |
| **Glucose** |
| **Erythrocyte sedimentation rate** |
| **Celiac serology**  Tissue transglutaminase antibodies  Immunoglobulin A  Endomysial antibodies |
| **Thyroid**  Free Thyroxine (fT4)  Triiodothyronine (T3)  Thyroid stimulating hormone  Thyroid binding globulin  Thyroid stimulating hormone receptor antibodies |
| **Vitamins**  Vitamin B12  Vitamin D  Vitamin D3  Vitamin B1  Vitamin B2  Vitamin B6  Vitamin C |
| **Iron status**  Iron  Iron saturation  Total iron capacity  Ferritin |
| **Antibodies indicating food allergy**  Peanut  Soy  Hazelnut  Almond  Crab  Shrimp  Tomato  Carrot  Orange  Potato  Coconut  Mussel  Tuna  Salmon  Strawberry  Avocado  Pecan  Cashew  Plum |
| **Hemoglobin A1c** |
| **Serology**  *Helicobacter pylori*  *Bordetella pertussis*  *Chlamydia trachomatis*  Epstein–Barr virus  *Bartonella henselae*  *Campylobacter* |
| **Urine tests** |
| **Urinalysis**  Protein  Glucose  Ketones  Nitrite  Leukocytes  pH  Erythrocytes  Bilirubin  Urobilinogen |
| **Urine dipslide** |
| **Urine other**  Human chorion gonadotrophin hormone (pregnancy test)  Creatinine  Albumin  24-hour protein  Urine occult blood test |
| **Fecal tests** |
| **Fecal calprotectin** |
| **Fecal occult blood test** |
| **Medicine prescriptions** |
| **Medication for non-acute abdominal pain and/or diarrhea** |
| **Laxatives** |
| Polyethylene glycol (macrogol) |
| Osmotic laxatives (lactulose) |
| Bulking agents  Psylla seeds  Sterculia gum |
| Enemas |
| Bisacodyl |
| Other laxatives |
| **Drugs for acid related disorders** |
| Proton pump inhibitors  Omeprazole  Esomeprazole  Pantoprazole |
| H_2_-receptor-antagonists  Ranitidine  Cimetidine |
| Antacids  Algeldrate/magnesium hydroxide  Calcium carbonate/magnesium carbonate  Alginic acid/sodium hydrogen carbonate/calcium carbonate |
| **Anti-infective agents** |
| Metronidazole |
| Mebendazole |
| Albendazole |
| Clioquinol |
| Fluconazole |
| Nitrofurantoin |
| **Diarrhea and vomiting inhibitors** |
| Vomiting inhibitors  Ondansetron  Metoclopramide  Domperidone |
| Diarrhea inhibitors  Loperamide  Norit (activated charcoal) |
| **Antispasmodics** |
| Mebeverine |
| Buscopan |
| Peppermint oil |
| **Miscellaneous** |
| Oral rehydration solution |
| Probiotics |
| **Medication manually checked for indication** |
| **Analgesics** |
| Paracetamol |
| Non-steroidal anti-inflammatory drugs (NSAID)  Ibuprofen  Diclofenac  Naproxen  Voltaren |
| Tramadol |
| **Miscellaneous** |
| Antibiotics other than nitrofurantoin, metronidazole,  mebendazole, albendazole, clioquinol, and fluconazole |
| Vitamin D |
| Ferrous fumarate |
| Magnesium |
| Rizatriptan |
| Melatonin |
| Contraception |

Supplementary Material S5. Supplementary tables of diagnostic testing and medicine prescriptions

| **Diagnostic tests** | **Children with ≥1 diagnostic test, n = 709** |
| --- | --- |
| **Blood tests** |  |
| C-reactive protein | 422 |
| Leukocytes  Eosinophils  Monocytes  Basophils  Leukocytes differentiation | 382  170  169  168  54 |
| Hemoglobin  Mean corpuscular volume  Erythrocytes  Hematocrit  Mean corpuscular hemoglobin  Mean corpuscular hemoglobin concentration  Red blood cell distribution width  Anemia diagnostics | 367  360  306  245  231  207  164  4 |
| Creatinine | 246 |
| Glucose | 206 |
| Erythrocyte sedimentation rate | 202 |
| Thyroid stimulating hormone | 201 |
| Thrombocytes  Shape abnormalities | 179  1 |
| Immunoglobulin A | 140 |
| Tissue transglutaminase antibodies | 126 |
| Vitamin B12 | 58 |
| Vitamin D | 48 |
| Ferritin | 39 |
| Free thyroxine | 32 |
| Food allergy | 26 |
| Epstein–Barr virus serology | 22 |
| Iron | 10 |
| Iron saturation | 7 |
| Total iron capacity | 7 |
| Vitamin B1 | 6 |
| Vitamin B6 | 6 |
| Endomysial antibodies | 3 |
| Hemoglobin A1c | 2 |
| *Helicobacter pylori* serology | 1 |
| **Urine tests** |  |
| Urinalysis | 244 |
| Urine dipslide | 79 |
| Urine other | 3 |
| Pregnancy test | 2 |
| **Fecal tests** |  |
| Fecal calprotectin | 7 |

| **Medication** | **Children with ≥1 medicine prescription, n = 760** |
| --- | --- |
| **Laxatives** |  |
| Polyethylene glycol (macrogol) | 468 |
| Osmotic laxatives (lactulose) | 52 |
| Bulking agents (psylla seeds) | 50 |
| Enemas | 17 |
| Bisacodyl | 8 |
| Other laxatives | 2 |
| **Drugs for acid related disorders** |  |
| Proton pump inhibitor | 55 |
| H_2_-receptor-antagonist | 16 |
| Antacids | 8 |
| **Anti-infective agents** |  |
| Nitrofurantoin | 26 |
| Other antibiotics | 22 |
| Metronidazole/mebendazole/albendazole/clioquinol/fluconazole | 7 |
| **Analgesics** |  |
| Non-steroidal anti-inflammatory drug (NSAID) | 26 |
| Paracetamol | 9 |
| **Diarrhea and vomiting inhibitors** |  |
| Domperidone | 11 |
| Diarrhea inhibitors | 8 |
| Metoclopramide | 7 |
| Ondansetron | 3 |
| **Antispasmodics** |  |
| Mebeverine | 15 |
| Buscopan | 5 |
| Peppermint oil | 2 |
| **Miscellaneous** |  |
| Contraception | 16 |
| Oral rehydration solution | 3 |
| Vitamin D | 6 |
| Ferrous fumarate | 4 |
| Magnesium | 2 |
| Rizatriptan | 1 |
| Probiotics | 2 |
| Melatonin | 1 |
